# Comparison of catastrophic out-of-pocket medical expenditure among older adults in the United States and South Korea: what affects the apparent difference?

**DOI:** 10.1186/s12913-022-08575-1

**Published:** 2022-09-26

**Authors:** Narae Kim, Mireille Jacobson

**Affiliations:** 1 University of Southern California Leonard Davis School of Gerontology, 3715 McClintock Ave., Los Angeles, California 90089 USA; 2 Leonard D. Schaeffer Center for Health Policy & Economics, 635 Downey Way Verna & Peter Dauterive Hall (VPD), Los Angeles, California 90089 USA

**Keywords:** Health insurance, Health care costs, Access to care, Catastrophic medical expenditures

## Abstract

**Background:**

Medical spending rises sharply with age. Even with universal health insurance, older adults may be at risk of catastrophic out-of-pocket medical spending. We aimed to compare catastrophic out-of-pocket medical spending among adults ages 65 and older in the United States, where seniors have near-universal coverage through Medicare, versus South Korea, where all residents have national health insurance.

**Methods:**

We used the 2016 Health and Retirement Study and the Korean Longitudinal Study of Aging. The study population were adults ages 65 and over in the US (*n* = 9,909) and South Korea (*n* = 4,450; *N* = 14,359). The primary outcome of interest was older adults’ exposure to catastrophic out-of-pocket medical expenditure, defined as out-of-pocket medical spending over the past two years that exceeded 50% of annual household income. To examine the factors affecting catastrophic out-of-pocket medical spending of older adults in both countries, we performed logistic regression analyses. To compare the contribution of demographic factors versus health system-level factors to catastrophic out-of-pocket medical spending, we performed a Blinder-Oaxaca decomposition.

**Results:**

The proportion of respondents with catastrophic out-of-pocket medical expenditure was 5.8% and 3.0% in the US and South Korea, respectively. A Blinder-Oaxaca decomposition showed that the difference in the rate of catastrophic out-of-pocket medical expenditure spending between the two countries was attributable largely to unobservable system-level factors, rather than observed differences in the sociodemographic characteristics.

**Conclusions:**

Exposure to catastrophic out-of-pocket medical spending is considerably higher in the US than South Korea. Most of the difference can be attributed to unobserved health system-level factors.

**Supplementary Information:**

The online version contains supplementary material available at 10.1186/s12913-022-08575-1.

## Background

The United States (US) spends twice as much per capita on health care as other industrialized countries [1]. While utilization accounts for some of the difference in health care spending, prior work suggests that prices account for the bulk of the difference [1–4]. Higher prices result from a mix of system-level factors that contribute to a shortage of health care resources, overpriced labor, goods and services, and high administrative costs [1–4]. Perhaps not surprisingly, the cost of health care is consistently rated among the most pressing concerns of Americans [5].

Less well-known is that, on average, Americans contribute less out-of-pocket to health care spending than their peers in other high-income nations [6]. This is true both as a share of total health care spending and in the purchasing power parity adjusted level of total out-of-pocket health care spending per capita [7]. However, these comparisons are only based on average out-of-pocket health care spending and do not reflect the fact that health care spending is highly skewed. In the US, for example, the top 20% of spenders in any year account for 80% of spending in the US [8]. Thus, average out-of-pocket spending may not adequately capture whether Americans are exposed to catastrophic out-of-pocket medical expenditures more or less frequently than in peer nations.

To date, relatively few studies have examined catastrophic out-of-pocket medical spending in the US, especially in comparison to other high-income countries. During the past decade, only four studies – Macinko [9] and Baird [10–12] – have explored the topic. Those studies demonstrate a positive correlation across countries between exposure to catastrophic medical spending and being poor, sick, disabled or aged. However, limited attention has been paid to older populations, who tend to be poorer and sicker than their younger counterparts and are, consequently, more exposed to the risk of catastrophic out-of-pocket medical spending [9]. In addition, factors affecting variation in older adults’ exposure across countries has not yet been explored.

To address the gap in the literature, we studied catastrophic out-of-pocket medical spending in the US and South Korea, specifically focusing on older adults, defined as those ages 65 and older. South Korea is a high-income country with national health insurance that is often overlooked in cross-country comparisons. The South Korean health care system offers an important contrast to the US health care system. Even though older adults ages 65 and over in both countries are covered by national health insurance, the South Korean national health insurance system is not very generous relative to other counties with universal national health insurance, offering more limited benefits and higher cost-sharing [13, 14]. Recent research has raised specific concerns about high out-of-pocket medical spending among poor and near-poor households in South Korea [15, 16]. Thus, at least in principle, South Korean older adults would seem to have more exposure to catastrophic out-of-pocket medical spending than their American counterparts.

In this study, we first examined factors that increase the odds of catastrophic out-of-pocket medical expenditures, separately in the US and South Korea. In particular, we considered age, gender, education, marital status and family composition, health status, health behaviors and supplemental health insurance. Second, we employed a Blinder-Oaxaca decomposition, a method commonly used in labor economics to understand wage differences across groups, to examine the impact of between-country differences in the risk of catastrophic out-of-pocket expenditures. Of key interest was whether differences in the likelihood of catastrophic out-of-pocket medical spending could be attributed to observable differences in age, health behaviors, self-rated health, or education versus health care system-level factors.

## Methods

### Data sources and sample population

We used data from the 2016 version of the Health and Retirement Study (HRS), processed and provided by RAND Corporation, and the Harmonized Korean Longitudinal Study of Aging (KLoSA). The HRS is a longitudinal study started in 1992 in the US with a nationally representative 9,267 households – 15,497 individuals – aged 51 and older recruited using a multistage probability sampling. Surveys are conducted every other year, and the survey population is refreshed every three waves. The surveys contain comprehensive information on demographics, health, cognition, family structure, housing, job status and history, health care use and costs, assets, and income [17].

KLoSA is a longitudinal study started in 2006 in South Korea. KLoSA’s initial survey was fielded to a nationally representative sample of 10,254 individuals aged 45 and older who were randomly selected to serve each enumeration district from the National Statistical Office’s 2005 Census data [18]; in 2014, 920 new respondents born between 1962 and 1963 were added to the original survey population. As a part of HRS-family studies, KLoSA provides information on health, socioeconomic status, and lifestyle of older adults in South Korea that is comparable to that in the HRS [19]. The harmonized version of KLoSA provided by Gateway to Aging has variables coded as identically as possible to those of the RAND HRS to enable international comparisons [20].

We limited our study sample to adults ages 65 and over to focus on a cross-country comparison of older adults. After excluding extreme outliers from the HRS and KLoSA, the total sample included 14,359 respondents – 9,909 from the HRS and 4,450 from KLoSA. Every participant in the study was covered by nationally provided health insurance, and approximately 86% of participants – 85.6% in the US and 87.8% in South Korea, respectively – paid out-of-pocket for medical services at least once between 2014 and 2016.

### Definition of catastrophic out-of-pocket medical expenditure

Catastrophic out-of-pocket medical expenditure is out-of-pocket spending on health care or medical services that exceeds one’s capacity to pay. Generally, high exposure to catastrophic out-of-pocket expenditure is closely related to low income, lack of financial risk protection (e.g., health insurance) and sick households [21, 22]. The incidence of households’ catastrophic out-of-pocket medical expenditures is observed all across the countries; however, low to middle-income countries show a higher incidence compared to high-income countries [21, 22]. Although the World Health Organization defines catastrophic out-of-pocket medical expenditures as out-of-pocket spending that exceeds 40% percent of a household’s “non-subsistence” income, studies often use varying percentages of income from 5% to 40% [15, 22]. In this study, we effectively used a 25% threshold as recommended in previous literature [9, 23]. As the HRS and KLoSA both report out-of-pocket health care spending from the two previous consecutive years, we coded individuals who had out-of-pocket health care spending during the last two years that exceeded 50% of annual household income as experiencing catastrophic out-of-pocket medical expenditures. As a sensitivity check, we also examined a 20% threshold, which was double another commonly used threshold – 10% [23].

### Statistical analysis

We first compared the observable characteristics of older adults in the US and South Korea and examined whether any differences were statistically different from each other. We performed logistic regression analyses with the HRS and KLoSA separately to examine the factors that affect the odds of catastrophic out-of-pocket medical expenditure in each country. The key outcome was a binary indicator of out-of-pocket medical spending over the previous two years that exceeded 50% of annual household income. Explanatory variables were age, gender, education, marital status, number of living children, ever diagnosed with high blood pressure, diabetes, cancer, lung disease, heart disease, stroke, psychiatric problem or arthritis, Body Mass Index (BMI), smoking history and whether the respondent had any supplemental health insurance.

To examine whether observable differences in population characteristics between the two countries accounted for the different risks of catastrophic out-of- pocket medical expenditure, we conducted a Blinder-Oaxaca decomposition. The Blinder-Oaxaca decomposition is a method first proposed by Blinder (1973) and Oaxaca (1973) to study differences in wages by race and gender [24–26]. Prior to this method, the difference in the intercepts from a wage equation for low earners (e.g., females) and high earners (e.g., males) was interpreted as discrimination. Blinder (1973) contended that the differences in coefficients were also affected by discrimination and developed an alternate structural equation to separate the "differences attributable to the differential in endowments" – observable characteristics – from the "differences attributable to the differential in coefficients" – unobservable discriminating factors [24, 26]. This method has been used more recently by health services researchers to study racial or educational disparities in health outcomes [27–29]. In the current context, we used the method to separate out between-country differences in catastrophic out-of-pocket medical spending due to observable sociodemographic differences, such as age and education (i.e., endowment differences), from differences in coefficients (i.e., system level factor differences). We included in the model only sociodemographic characteristics and a few health-related variables that were plausibly not affected by differences in the health systems of the two countries. Specifically, we adjusted for age, gender, education, marital status, number of living children, income, self-rated health, BMI and smoking history. As other health-related variables such as medical conditions and health care utilizations influence and are influenced by the outcome of interest – exposure to catastrophic out-of-pocket medical expenditure – we let the model reflect those as system-level factors by excluding those from the model.

All the analyses were performed with Stata/MP 16.1. All the statistical results were considered significant at a confidence level of 95%.

## Results

The final study sample included 14,359 individuals – 9,909 from the HRS and 4,450 from KLoSA. The mean age of respondents in the US sample was 75.96 and in the Korean sample was 75.67. Significant differences were found in observable characteristics of the two groups and were most notable for educational attainment and health history. A higher share of US seniors had graduated high school or college and had supplemental health insurance compared to their South Korean counterparts. Although US seniors more positively assessed their health, they had a higher probability of being diagnosed with high blood pressure, diabetes, cancer, lung disease, heart problem, stroke, psychiatric problem, or arthritis; were more obese; and were more likely to have ever smoked (Table 1).

**Table 1:** Descriptive Statistics of Respondent Age 65 and Older

	United States	South Korea	*ρ*
N	9,909	4,450	
Age (mean, %)	75.96	75.67	0.031* 0.007**
65–74	45.17	46.94	
75–84	40.12	40.25	
85 +	14.71	12.81	
Gender (%)			0.041
Female	59.4	57.58	
Education (%)			< 0.001
Less than high school	19.29	72.43	
High school graduate	34.62	20.22	
College and above	46.09	7.35	
Marital Status (%)			< 0.001
Married or living with a partner	57.65	68.67	
Not Married	42.35	31.33	
Number of living children (mean)	3.24	3.28	
Disease (%)			
High Blood Pressure	71.13	53.67	< 0.001
Diabetes	30.36	24.16	< 0.001
Cancer	21.58	7.31	< 0.001
Lung disease	12.76	4.31	< 0.001
Heart problem	34.05	12.54	< 0.001
Stroke	12.79	8.16	< 0.001
Psychiatric problem	20.67	5.75	< 0.001
Arthritis	71.96	34.63	< 0.001
Self-rated Health (%)			< 0.001
Excellent	6.72	0.13	
Very good	26.82	3.30	
Good	34.65	25.91	
Fair	23.32	41.33	
Poor	8.48	29.33	
BMI (mean)	27.93	23.18	< 0.001
Smoking history (%)	55.02	30.45	< 0.001
Have supplemental health insurance (%)	41.31	16.74	< 0.001

^*^ρ value for two-sample t tests for means

^**^ρ value for Pearson’s chi-squared tests for proportions

**Fig. 1:**
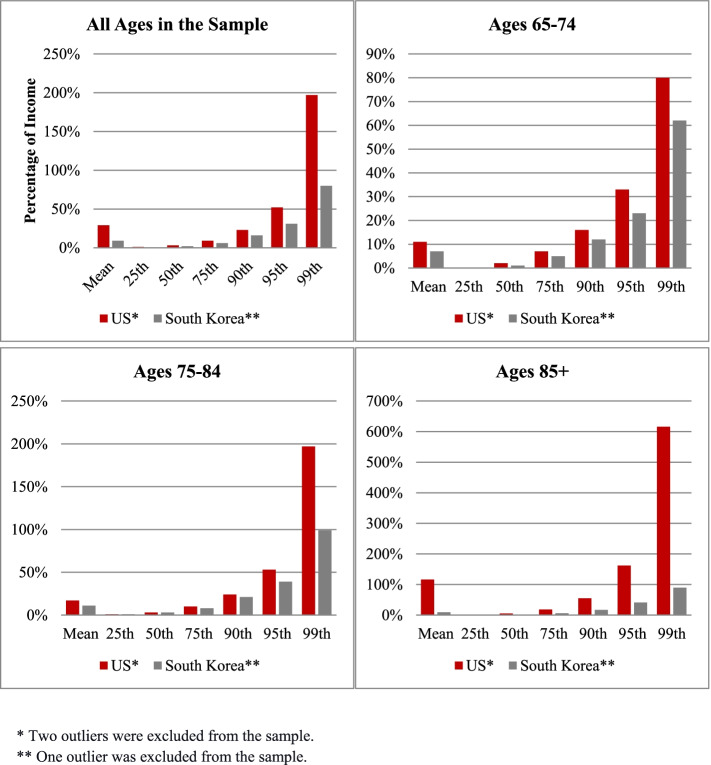
Distribution of Out-of-Pocket Heatlh Care Spending Relative to Income in the United States and South Korea Overall and by Age Group

**Fig. 2:**
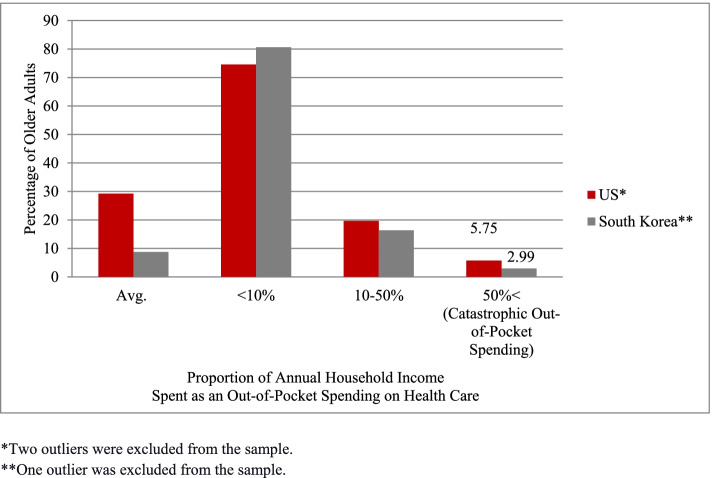
Percentage of Older Adults in Different Categories of the Proportion of Annual Household Income Spent as an Out-of-Pocket Health Care Spending

Compared to those in South Korea, older adults in the US spent a higher proportion of annual household income on out-of-pocket medical expenditure (Fig. 1). At the mean, older adults in the US spent about 29% of their annual household income on out-of-pocket health care spending over the prior two years; older adults in South Korea spent nearly 9% of income on out-of-pocket health care spending over the prior two years. The higher proportion in the US versus South Korea is observed not only at the mean but also at all points in the distribution of medical spending over the past two years relative to household income and across all age groups. The most notable difference was for the oldest group. Among those ages 85 and over, the ratio of out-of-pocket medical spending in the past two years was 1.16 in the US but only 0.09 in South Korea. This stark difference is driven by the tails of the distribution. In the US, the average proportion for those in the 90th percentile of the oldest group was 0.55 or 55% of annual household income, implying that this group faces catastrophic out-of-pocket medical expenditure, while in South Korea, the 90^th^ percentile was 17% of annual income on out-of-pocket medical spending. Overall in the US, 5.75% of older adults had catastrophic out-of-pocket health care spending, meaning spending over the past two years that exceeded 50% of annual household income, while in South Korea, only 2.99% did (Fig. 2). The same finding that the US had higher exposure to catastrophic out-of-pocket medical expenditures than South Korea was observed across subgroups of older adults as defined by 1) supplemental health insurance status, 2) common chronic medical conditions or 3) history of hospitalization (Appendix Figure 1).

9,247 and 3,832 participants from the HRS and KLoSA, respectively, were included in the logistic regressions after excluding those with missing covariates. Logistic regression analyses showed that age, marital status, diabetes, cancer, stroke, psychiatric problems, BMI, and income were statistically significantly correlated with the odds of exposure to catastrophic out-of-pocket medical expenditure in the HRS. Those who were older; were not married; had diabetes, cancer, stroke or psychiatric problem; had higher BMI, and had lower income had higher odds of catastrophic out-of-pocket medical expenditure. In KLoSA, those who were college educated; had diabetes, cancer, stroke or arthritis, and had lower income had higher odds of catastrophic out-of-pocket medical expenditure (Table 2). Logistic regression analyses with a 20% threshold showed qualitatively similar patterns (Appendix Table 1).

**Table 2:** Odds of Being Exposed to Catastrophic Out-of-Pocket Health Care Spending in the United States and South Korea

	United States			South Korea		
N	9,247			3,832		
	OR	SE	*ρ*	OR	SE	*ρ*
Age						
65–74 (ref)						
75–84	1.419	0.175	0.005	1.101	0.259	0.682
85 +	2.472	0.345	< 0.001	1.262	0.405	0.469
Female	1.162	0.137	0.205	1.103	0.333	0.745
Education						
Less than high school (ref)						
High school graduate	1.180	0.149	0.190	1.102	0.341	0.753
College and above	1.365	0.183	0.020	3.636	1.361	< 0.001
Marital Status						
Married (ref)						
Not married	1.560	0.187	< 0.001	1.099	0.232	0.656
Number of living children	0.977	0.022	0.285	0.939	0.059	0.314
Income Quartiles						
25% (ref)						
50%	0.404	0.046	< 0.001	0.227	0.0594	< 0.001
75%	0.141	0.028	< 0.001	0.019	0.0189	< 0.001
100%	0.059	0.022	< 0.001	1	-	-
Diseases						
High blood pressure	1.104	0.141	0.437	1.307	0.284	0.219
Diabetes	1.228	0.130	0.053	1.494	0.300	0.045
Cancer	1.161	0.132	0.190	1.832	0.527	0.035
Lung disease	1.111	0.144	0.417	1.070	0.426	0.864
Heart problem	1.069	0.112	0.523	1.322	0.307	0.230
Stroke	2.008	0.228	< 0.001	1.714	0.446	0.039
Psychiatric problem	1.399	0.153	0.002	0.895	0.318	0.755
Arthritis	0.856	0.105	0.207	1.821	0.381	0.004
Self-rated Health						
Excellent (ref. in HRS)				1.000	-	-
Very good	1.093	0.361	0.788	0.539	0.403	0.409
Good	1.619	0.511	0.126	0.304	0.105	< 0.001
Fair	2.742	0.864	0.001	0.518	0.113	0.003
Poor (ref. in KLoSA)	3.852	1.261	< 0.001	1.000	*Omitted*	*Omitted*
BMI	0.981	0.008	0.027	1.025	0.0317	0.422
Smoking History	1.105	0.113	0.330	0.765	0.221	0.354
Supplemental Health Insurance	1.211	0.127	0.069	1.474	0.480	0.234
R Squared			0.1881			0.1793

*OR* refers to Odds Ratio, *SE* refers to Standard Error, *BMI* refers to Body Mass Index

The Blinder-Oaxaca decomposition showed a statistically significant difference in the risk of catastrophic out-of-pocket medical spending between the two countries. Notably, the majority of the difference was driven by unobservable systemic factors, not by observable population characteristics. After adjusting for the demographic and socioeconomic characteristics of older adults in South Korea, only 14.8% of the difference in the probability of catastrophic out-of-pocket medical spending in the US was explained. When including self-rated health and behavioral variables, specifically BMI and smoking history, in the model, the explainable difference increased to 15.4%, implying that differences in health behaviors across the two countries contribute to only a small share of the overall difference (Table 3). As a robustness check, multiple analyses were performed with randomized order of the variables; the analyses showed the same basic result, confirming that the finding was not sensitive to the order of the included variable. The Blinder-Oaxaca decomposition with a 20% threshold also showed a statistically significant difference between the two countries with more weight on systemic differences (Appendix Table 2). As another sensitivity analysis, health conditions were included in the Blinder-Oaxaca decomposition. In contrast to the main finding, this decomposition showed that the majority of the difference in catastrophic health spending was explained by observable differences (i.e., differences in health conditions) (Appendix Table 3). However, since disease incidence is a function of both the health system (e.g., the prevalence of specific health screenings) and underlying population health, it is difficult to separately attribute the additional “explained” portion of the difference in catastrophic spending to the health system versus underlying health.

**Table 3:** Decomposition for Probability of Being Exposed to Catastrophic Out-of-Pocket Health Care Spending of the United States and South Korea

	Logistic Regression Estimates			Decomposition Estimates			
				Without Health & Behavioral Variables		With Health & Behavioral Variables	
	OR	SE	ρ	Absolute Difference	Relative Proportion	Absolute Difference	Relative Proportion
South Korea	0.327	0.042	< 0.001				
Age				0.000	0.027	0.000	0.018
65–74 (ref)				0.000		0.000	
75–84	1.37	0.143	0.003	0.000		0.000	
85 +	2.295	0.274	< 0.001	0.000		0.000	
Gender (female)	1.172	0.121	0.124	0.000	0.037	0.000	0.012
Education				0.010	2.802	0.012	3.318
Less than high school (ref)				0.014		0.016	
High school graduate	1.257	0.140	0.0398	-0.004		-0.004	
College and above	1.581	0.191	< 0.001	0.000		0.000	
Marital Status				0.001	0.153	0.001	0.155
Married (ref)				0.001		0.001	
Not married	1.460	0.148	< 0.001	0.000		0.000	
Number of living children	0.963	0.020	0.064	0.000	0.024	0.000	0.021
Income Quartiles				-0.008	-2.038	-0.007	-1.931
25% (ref)				-0.008		-0.007	
50%	0.363	0.037	< 0.001	0.000		0.000	
75%	0.117	0.022	< 0.001	0.000		0.000	
100%	0.043	0.016	< 0.001	0.000		0.000	
Self-rated Health						-0.011	-3.076
Excellent (ref)						-0.003	
Very good	1.145	0.374	0.678			0.000	
Good	1.817	0.564	0.054			-0.001	
Fair	3.377	1.037	< 0.001			0.000	
Poor	5.964	1.861	< 0.001			-0.007	
BMI	0.985	0.008	0.059			0.011	2.901
Smoking history	1.088	0.099	0.355			-0.002	-0.414
Total explained				0.004		0.004	
Unexplained*				0.023		0.023	
Total difference*				0.027	1.000	0.026	1.000
R Squared			0.1750				

Absolute differences were obtained from coefficients of endowments of each covariate which estimate how between-country difference in each covariate accounted for the overall difference in exposure to catastrophic out-of-pocket medical expenditures between the two countries

^*^ *ρ* < 0.001

## Discussion

In this work, we compared catastrophic out-of-pocket medical spending among adults ages 65 and older in the United States and South Korea. We focused on older adults because this demographic faces a disproportionate burden of disease and, relatedly, constitutes a disproportionate share of overall medical spending [30, 31]. In addition, prior work on catastrophic health spending has not isolated impacts on this group.

The study findings demonstrate that older adults in the US had a significantly higher risk of catastrophic out-of-pocket medical spending compared to South Korea; nearly 6% of seniors in the HRS reported that they spent more than 50% of their annual household income on health care in the past two years. Sociodemographic characteristics and health history were found to affect the differences in odds across countries; however, the difference was not primarily attributable to observable sociodemographic but to unobservable systemic factors. Despite near universal Medicare coverage, older adults in the US face considerably higher out-of-pocket medical spending risks than their South Korean counterparts.

Systemic differences identified in the decomposition may be attributable to many different things. Using health care utilization data from the HRS and KLoSA, we found a significant difference in inpatient and outpatient service utilization during the past two years in the US relative to South Korea. In the US, older adults tend to use more inpatient services, while in South Korea, older adults tend to use more outpatient services (Appendix Figure 2). The difference in utilization between the two countries was the most significant among the oldest and those in the lowest income quartile. As the oldest and the poorest in the US were the most vulnerable to catastrophic out-of-pocket medical expenditure, higher inpatient service utilization and lower outpatient service utilization likely contribute to higher catastrophic out-of-pocket medical expenditure risk of the US. Another important contributing factor may be differences in the cost-sharing for expensive health care services. South Korea’s coverage of certain high-cost services is actually more comprehensive than the United States. For example, while the Korean national health insurance covers a hospital stay for an unlimited period with 20% of coinsurance, Medicare only covers the first 60 days after an initial deductible has been paid ($1,556 in 2022), and then days 60 to 90 are subject to daily coinsurance ($389 per day in 2022) [32, 33]. The proportion of people younger than 65 with health insurance is another major systemic difference between the two countries. In South Korea, all residents are covered by national health insurance from birth, while in the US, a significant proportion of people under 65 lack health insurance. In 2016, 10.0% of the nonelderly population or 26.7 million people were uninsured in the US [34]. Since the uninsured are more likely to delay medical care until they become eligible for Medicare at age 65, they may be at increased risk of catastrophic out-of-pocket medical expenditure in later life [35]. In addition, as South Korea is under a single-payer system, health care prices are actively managed and thus systematically lower than those in the US. Since the decomposition itself does not clarify the source of the disparity, future studies are needed to pin down the specific contributors to differences in catastrophic medical spending among older adults.

The study has several limitations. First, differences in screening and treating pre-existing health conditions between the two countries could bias the results. When comparing the results of logistic regressions separately conducted based on the HRS and the KLoSA datasets, diagnosed health conditions affected the out-of-pocket expenditure differently in the two countries. While having psychiatric problems significantly positively affected the expenditure in the US, it was found to be a statistically insignificant factor that negatively affected expenditures in South Korea. This could be due to the fact that psychiatric counselling and diagnosis are less common in South Korea [36]. Similarly, the diagnosis of cancer was found to be a statistically insignificant factor in the US; however, it was one of the driving factors of high out-of-pocket expenditure in South Korea. This difference also could be due to the high screening rate of cancer (e.g., thyroid) in South Korea [37].

Another limitation stemmed from the possibility of measurement error. As the expenditure data came from the survey, it was subject to recall bias. In addition, the out-of-pocket expenditure used in this study did not include the costs for medical devices (e.g., wheelchairs) due to the lack of comparable variables across surveys. Thus, this study missed a potentially important source of out-of-pocket health care spending.

## Conclusions

The study shows a high risk of catastrophic out-of-pocket health expenditure facing older adults in the US and confirms that the proportion is considerably higher than in South Korea, a comparable developed country that also offers national health insurance to older adults. The Blinder-Oaxaca decomposition showed that the higher risk is attributed primarily to systemic factors, not observable socioeconomic or demographic differences. However, this method cannot pinpoint the specific systemic factors that cause this difference. The possible systemic factors that contribute to the difference include differences in the generosity of cost-sharing, overall health care prices and subsequent utilization. Recent legislative changes passed under the Inflation Reduction Act tackle several of these issues for older adults – capping out-of-pocket prescription drug costs for individuals on Medicare and allowing Medicare to negotiate prices for some prescription drugs [38]. Future work will be needed to understand whether these changes reduce catastrophic out-of-pocket medical spending among older adults in the US and narrow differences between the US and South Korea.

## Supplementary Information

**Additional file 1: Figure 1.** Percentage of Older Adults in Different Categories of the Proportion of Annual Household Income Spent as an Out-of-Pocket Spending on Health Care by Various Subgroups.

**Additional file 2: Figure 2.** Average Inpatient & Outpatient Service Utilization of Two Years by Age Categories and Income Quartiles in the US and South Korea.

**Additional file 3: Table 1.** Odds of Being Exposed to Catastrophic Health Spending in the US and South Korea with 20% threshold.

**Additional file 4: Table 2.** US-South Korea Decomposition for Probability of Being Exposed to Catastrophic Out-of-Pocket Medical Expenditure with 20% Threshold.

**Additional file 5: Table 3.** US-South Korea Decomposition with Health Conditions Variables for Probability of Being Exposed to Catastrophic Out-of-Pocket Medical Expenditure.

## Data Availability

The datasets used for this research are publicly available from the following links: HRS—https://hrsdata.isr.umich.edu/ KLoSA- https://survey.keis.or.kr/eng/

## Abbreviations

US: United States
HRS: Health and Retirement Study
KLoSA: Korean Longitudinal Study of Aging
BMI: Body Mass Index
