# Supplementary material for: Comparison of catastrophic out-of-pocket medical expenditure among older adults in the United States and South Korea: what affects the apparent difference?
Source: BMC Health Serv Res. 2022 Sep 26;22:1202. doi: 10.1186/s12913-022-08575-1 (PMC9511719; doi:10.1186/s12913-022-08575-1)
Supplement: Supplementary file 1 — Additional file 1: Figure 1. Percentage of Older Adults in Different Categories of the Proportion of Annual Household Income Spent as an Out-of-Pocket Spending on Health Care by Various Subgroups. [file 12913_2022_8575_MOESM1_ESM.docx]

Figure1. Percentage of Older Adults in Different Categories of the Proportion of Annual Household Income Spent as an Out-of-Pocket Spending on Health Care by Various Subgroups

2-1. Older Adults with Supplemental Health Insurance

*n=4,093; **n=745

2-2. Older Adults without Supplemental Health Insurance

*n=5,816; **n=3,705

2-3. Older Adults with High Blood Pressure

*n=7,024; n=2,388

2-4. Older Adults without High Blood Pressure

*n=2,852; **n=2,062

2-5. Older Adults with History of Hospitalization

*n=3,142; n=594

2-6. Older Adults without History of Hospitalization

*n=6,767; **n=3,856
