# Supplementary material for: Comparison of catastrophic out-of-pocket medical expenditure among older adults in the United States and South Korea: what affects the apparent difference?
Source: BMC Health Serv Res. 2022 Sep 26;22:1202. doi: 10.1186/s12913-022-08575-1 (PMC9511719; doi:10.1186/s12913-022-08575-1)
Supplement: Supplementary file 2 — Additional file 2: Figure 2. Average Inpatient & Outpatient Service Utilization of Two Years by Age Categories and Income Quartiles in the US and South Korea. [file 12913_2022_8575_MOESM2_ESM.docx]

Figure2. Average Inpatient & Outpatient Service Utilization of Two Years by Age Categories and Income Quartiles in the US and South Korea

Inpatient Outpatient

Inpatient Outpatient
