# Supplementary material for: Comparison of catastrophic out-of-pocket medical expenditure among older adults in the United States and South Korea: what affects the apparent difference?
Source: BMC Health Serv Res. 2022 Sep 26;22:1202. doi: 10.1186/s12913-022-08575-1 (PMC9511719; doi:10.1186/s12913-022-08575-1)
Supplement: Supplementary file 3 — Additional file 3: Table 1. Odds of Being Exposed to Catastrophic Health Spending in the US and South Korea with 20% threshold. [file 12913_2022_8575_MOESM3_ESM.docx]

Table1. Odds of Being Exposed to Catastrophic Health Spending in the US and South Korea with 20% threshold

|  | United States | | | South Korea | | |
| --- | --- | --- | --- | --- | --- | --- |
| N | 9,247 | | | 3,832 | | |
|  | OR | SE | ρ | OR | SE | ρ |
| Age |  |  |  |  |  |  |
| 65-74 (ref) |  |  |  |  |  |  |
| 75-84 | 1.232 | 0.095 | 0.007 | 1.035 | 0.143 | 0.803 |
| 85+ | 1.734 | 0.165 | 0.000 | 0.756 | 0.154 | 0.171 |
| Female | 1.100 | 0.084 | 0.210 | 0.905 | 0.159 | 0.570 |
| Education |  |  |  |  |  |  |
| Less than high school (ref) |  |  |  |  |  |  |
| High school graduate | 1.247 | 0.108 | 0.011 | 1.274 | 0.210 | 0.141 |
| College and above | 1.452 | 0.133 | 0.000 | 1.960 | 0.502 | 0.009 |
| Marital Status |  |  |  |  |  |  |
| Married (ref) |  |  |  |  |  |  |
| Not married | 1.219 | 0.092 | 0.009 | 1.042 | 0.136 | 0.753 |
| Number of living children | 0.976 | 0.015 | 0.108 | 1.012 | 0.039 | 0.755 |
| Income Quartiles |  |  |  |  |  |  |
| 25% (ref) |  |  |  |  |  |  |
| 50% | 0.556 | 0.042 | 0.000 | 0.270 | 0.038 | 0.000 |
| 75% | 0.198 | 0.022 | 0.000 | 0.076 | 0.021 | 0.000 |
| 100% | 0.057 | 0.012 | 0.000 | 0.035 | 0.018 | 0.000 |
| Diseases |  |  |  |  |  |  |
| High blood pressure | 1.174 | 0.096 | 0.050 | 1.305 | 0.163 | 0.033 |
| Diabetes | 1.118 | 0.080 | 0.119 | 1.503 | 0.184 | 0.001 |
| Cancer | 1.008 | 0.079 | 0.917 | 1.953 | 0.353 | 0.000 |
| Lung disease | 1.128 | 0.101 | 0.182 | 1.405 | 0.323 | 0.139 |
| Heart problem | 1.135 | 0.079 | 0.069 | 1.957 | 0.271 | 0.000 |
| Stroke | 1.574 | 0.132 | 0.000 | 1.298 | 0.225 | 0.132 |
| Psychiatric problem | 1.247 | 0.095 | 0.004 | 0.923 | 0.199 | 0.710 |
| Arthritis | 1.070 | 0.087 | 0.405 | 1.445 | 0.178 | 0.003 |
| Self-rated Health |  |  |  |  |  |  |
| Excellent (ref. in HRS) |  |  |  | 1.000 | - | - |
| Very good | 1.009 | 0.192 | 0.963 | 0.507 | 0.200 | 0.085 |
| Good | 1.566 | 0.287 | 0.014 | 0.241 | 0.047 | 0.000 |
| Fair | 2.121 | 0.393 | 0.000 | 0.481 | 0.061 | 0.000 |
| Poor (ref. in KLoSA) | 2.753 | 0.549 | 0.000 | 1.000 | *Omitted* | *Omitted* |
| BMI | 0.986 | 0.006 | 0.018 | 0.995 | 0.019 | 0.792 |
| Smoking History | 0.957 | 0.065 | 0.515 | 1.015 | 0.166 | 0.927 |
| Supplemental Health Insurance | 1.133 | 0.079 | 0.074 | 1.256 | 0.239 | 0.230 |
| R Squared |  |  | 0.1512 |  |  | 0.2045 |
