# Supplementary material for: Comparison of catastrophic out-of-pocket medical expenditure among older adults in the United States and South Korea: what affects the apparent difference?
Source: BMC Health Serv Res. 2022 Sep 26;22:1202. doi: 10.1186/s12913-022-08575-1 (PMC9511719; doi:10.1186/s12913-022-08575-1)
Supplement: Supplementary file 4 — Additional file 4: Table 2. US-South Korea Decomposition for Probability of Being Exposed to Catastrophic Out-of-Pocket Medical Expenditure with 20% Threshold. [file 12913_2022_8575_MOESM4_ESM.docx]

Tabe2. US-South Korea Decomposition for Probability of Being Exposed to Catastrophic Out-of-Pocket Medical Expenditure with 20% Threshold

|  | Logistics | | |  | Decomposition Estimates | | | | | | |
| --- | --- | --- | --- | --- | --- | --- | --- | --- | --- | --- | --- |
|  |  |  |  |  | Without Health & Behavioral Variables | | |  | With Health & Behavioral Variables | | |
|  | OR | SE | ρ |  | Absolute Difference |  | Relative Proportion |  | Absolute Difference |  | Relative Proportion |
|  |  |  |  |  |  |  |  |  |  |  |  |
| South Korea | 0.428 | 0.035 | 0.000 |  |  |  |  |  |  |  |  |
| Age |  |  |  |  | 0.000 |  | 0.029 |  | -0.001 |  | 0.040 |
| 65-74 (ref) |  |  |  |  | 0.000 |  |  |  | -0.001 |  |  |
| 75-84 | 1.284 | 0.082 | 0.000 |  | 0.000 |  |  |  | 0.000 |  |  |
| 85+ | 1.646 | 0.134 | 0.000 |  | 0.000 |  |  |  | 0.000 |  |  |
| Gender (female) | 1.094 | 0.071 | 0.168 |  | 0.000 |  | -0.002 |  | 0.000 |  | 0.005 |
| Education |  |  |  |  | 0.013 |  | -1.630 |  | 0.020 |  | -0.867 |
| Less than high school (ref) |  |  |  |  | 0.016 |  |  |  | 0.023 |  |  |
| High school graduate | 1.355 | 0.099 | 0.000 |  | -0.003 |  |  |  | -0.003 |  |  |
| College and above | 1.607 | 0.130 | 0.000 |  | 0.000 |  |  |  | 0.000 |  |  |
| Marital Status |  |  |  |  | 0.001 |  | -0.168 |  | 0.001 |  | -0.035 |
| Married (ref) |  |  |  |  | 0.001 |  |  |  | 0.001 |  |  |
| Not married | 1.177 | 0.075 | 0.010 |  | 0.000 |  |  |  | 0.000 |  |  |
| Number of living children | 0.970 | 0.013 | 0.028 |  | 0.000 |  | 0.006 |  | 0.000 |  | 0.000 |
| Income Quartiles |  |  |  |  | -0.022 |  | 2.766 |  | -0.019 |  | 0.849 |
| 25% (ref) |  |  |  |  | -0.022 |  |  |  | -0.019 |  |  |
| 50% | 0.468 | 0.030 | 0.000 |  | -0.001 |  |  |  | -0.001 |  |  |
| 75% | 0.164 | 0.016 | 0.000 |  | 0.001 |  |  |  | 0.001 |  |  |
| 100% | 0.051 | 0.010 | 0.000 |  | 0.000 |  |  |  | 0.000 |  |  |
| Self-rated Health |  |  |  |  |  |  |  |  | -0.035 |  | 1.535 |
| Excellent (ref) |  |  |  |  |  |  |  |  | -0.009 |  |  |
| Very good | 1.118 | 0.210 | 0.555 |  |  |  |  |  | 0.000 |  |  |
| Good | 1.724 | 0.309 | 0.002 |  |  |  |  |  | -0.003 |  |  |
| Fair | 2.589 | 0.464 | 0.000 |  |  |  |  |  | -0.001 |  |  |
| Poor | 4.837 | 0.892 | 0.000 |  |  |  |  |  | -0.022 |  |  |
| BMI | 0.992 | 0.005 | 0.116 |  |  |  |  |  | 0.011 |  | -0.502 |
| Smoking history | 1.008 | 0.060 | 0.894 |  |  |  |  |  | 0.000 |  | -0.021 |
| Total explained |  |  |  |  | -0.008 |  |  |  | -0.023 |  |  |
| Unexplained* |  |  |  |  | 0.050 |  |  |  | 0.065 |  |  |
| Total difference* |  |  |  |  | 0.042 |  | 1.000 |  | 0.042 |  | 1.000 |
| R Squared |  |  | 0.1482 |  |  |  |  |  |  |  |  |

* ρ <0.001
