# Supplementary material for: Comparison of catastrophic out-of-pocket medical expenditure among older adults in the United States and South Korea: what affects the apparent difference?
Source: BMC Health Serv Res. 2022 Sep 26;22:1202. doi: 10.1186/s12913-022-08575-1 (PMC9511719; doi:10.1186/s12913-022-08575-1)
Supplement: Supplementary file 5 — Additional file 5: Table 3. US-South Korea Decomposition with Health Conditions Variables for Probability of Being Exposed to Catastrophic Out-of-Pocket Medical Expenditure. [file 12913_2022_8575_MOESM5_ESM.docx]

Table3. US-South Korea Decomposition with Health Conditions Variables for Probability of Being Exposed to Catastrophic Out-of-Pocket Medical Expenditure

|  | 50% Threshold | | | | | | |  | 20% Threshold | | | | | | |
| --- | --- | --- | --- | --- | --- | --- | --- | --- | --- | --- | --- | --- | --- | --- | --- |
|  | Logistics | | |  | Decomposition Estimates | | |  | Logistics | | |  | Decomposition Estimates | | |
|  | OR | SE | ρ |  | Absolute Difference |  | Relative Proportion |  | OR | SE | ρ |  | Absolute Difference |  | Relative Proportion |
|  |  |  |  |  |  |  |  |  |  |  |  |  |  |  |  |
| South Korea | 0.450 | 0.064 | 0.000 |  |  |  |  |  | 0.577 | 0.052 | 0.000 |  |  |  |  |
| Age |  |  |  |  | 0.000 |  | 0.002 |  |  |  |  |  | -0.001 |  | -0.056 |
| 65-74 (ref) |  |  |  |  | 0.000 |  |  |  |  |  |  |  | -0.001 |  |  |
| 75-84 | 1.342 | 0.144 | 0.006 |  | 0.000 |  |  |  | 1.211 | 0.079 | 0.003 |  | 0.000 |  |  |
| 85+ | 2.127 | 0.265 | 0.000 |  | 0.000 |  |  |  | 1.481 | 0.125 | 0.000 |  | 0.000 |  |  |
| Gender (female) | 1.189 | 0.128 | 0.107 |  | 0.000 |  | -0.002 |  | 1.077 | 0.073 | 0.273 |  | 0.000 |  | -0.013 |
| Education |  |  |  |  | 0.013 |  | 0.715 |  |  |  |  |  | 0.020 |  | 1.040 |
| Less than high school (ref) |  |  |  |  | 0.017 |  |  |  |  |  |  |  | 0.023 |  |  |
| High school graduate | 1.235 | 0.140 | 0.064 |  | -0.004 |  |  |  | 1.336 | 0.100 | 0.000 |  | -0.003 |  |  |
| College and above | 1.549 | 0.191 | 0.000 |  | 0.000 |  |  |  | 1.618 | 0.134 | 0.000 |  | 0.000 |  |  |
| Marital Status |  |  |  |  | 0.000 |  | 0.024 |  |  |  |  |  | 0.001 |  | 0.029 |
| Married (ref) |  |  |  |  | 0.000 |  |  |  |  |  |  |  | 0.001 |  |  |
| Not married | 1.396 | 0.144 | 0.001 |  | 0.000 |  |  |  | 1.132 | 0.073 | 0.054 |  | 0.000 |  |  |
| Number of living children | 0.966 | 0.020 | 0.095 |  | 0.000 |  | 0.004 |  | 0.973 | 0.014 | 0.054 |  | 0.000 |  | 0.000 |
| Income Quartiles |  |  |  |  | -0.007 |  | -0.383 |  |  |  |  |  | -0.019 |  | -1.000 |
| 25% (ref) |  |  |  |  | -0.007 |  |  |  |  |  |  |  | -0.018 |  |  |
| 50% | 0.365 | 0.038 | 0.000 |  | 0.000 |  |  |  | 0.464 | 0.030 | 0.000 |  | -0.001 |  |  |
| 75% | 0.115 | 0.022 | 0.000 |  | 0.000 |  |  |  | 0.161 | 0.016 | 0.000 |  | 0.000 |  |  |
| 100% | 0.046 | 0.017 | 0.000 |  | 0.000 |  |  |  | 0.049 | 0.010 | 0.000 |  | 0.000 |  |  |
| Self-rated Health |  |  |  |  | -0.008 |  | -0.440 |  |  |  |  |  | -0.025 |  | -1.336 |
| Excellent (ref) |  |  |  |  | 0.000 |  |  |  |  |  |  |  | 0.000 |  |  |
| Very good | 1.070 | 0.352 | 0.837 |  | 0.012 |  |  |  | 1.007 | 0.191 | 0.969 |  | 0.033 |  |  |
| Good | 1.460 | 0.458 | 0.228 |  | 0.004 |  |  |  | 1.369 | 0.249 | 0.084 |  | 0.009 |  |  |
| Fair | 2.401 | 0.749 | 0.005 |  | -0.009 |  |  |  | 1.868 | 0.342 | 0.001 |  | -0.024 |  |  |
| Poor | 3.762 | 1.205 | 0.000 |  | -0.015 |  |  |  | 3.111 | 0.593 | 0.000 |  | -0.043 |  |  |
| BMI | 0.980 | 0.008 | 0.016 |  | 0.006 |  | 0.334 |  | 0.983 | 0.005 | 0.002 |  | 0.000 |  | 0.000 |
| Smoking history | 1.033 | 0.097 | 0.730 |  | -0.002 |  | -0.096 |  | 0.969 | 0.060 | 0.605 |  | 0.000 |  | -0.014 |
| Diseases |  |  |  |  | 0.015 |  | 0.842 |  |  |  |  |  | 0.044 |  | 2.344 |
| High blood pressure | 1.187 | 0.130 | 0.118 |  | 0.001 |  |  |  | 1.229 | 0.083 | 0.002 |  | 0.003 |  |  |
| Diabetes | 1.276 | 0.119 | 0.009 |  | 0.001 |  |  |  | 1.205 | 0.074 | 0.002 |  | 0.003 |  |  |
| Cancer | 1.248 | 0.132 | 0.037 |  | 0.003 |  |  |  | 1.121 | 0.081 | 0.112 |  | 0.009 |  |  |
| Lung disease | 1.090 | 0.134 | 0.484 |  | 0.000 |  |  |  | 1.112 | 0.093 | 0.206 |  | 0.003 |  |  |
| Heart problem | 1.125 | 0.107 | 0.217 |  | 0.003 |  |  |  | 1.272 | 0.080 | 0.000 |  | 0.016 |  |  |
| Stroke | 1.967 | 0.203 | 0.000 |  | 0.001 |  |  |  | 1.516 | 0.114 | 0.000 |  | 0.002 |  |  |
| Psychiatric problem | 1.291 | 0.133 | 0.013 |  | -0.001 |  |  |  | 1.166 | 0.083 | 0.032 |  | -0.002 |  |  |
| Arthritis | 1.096 | 0.118 | 0.392 |  | 0.007 |  |  |  | 1.215 | 0.082 | 0.004 |  | 0.012 |  |  |
| Total explained |  |  |  |  | 0.018 |  | 1.000 |  |  |  |  |  | 0.019 |  | 1.000 |
| Unexplained* |  |  |  |  | 0.007 |  |  |  |  |  |  |  | 0.022 |  |  |
| Total difference* |  |  |  |  | 0.025 |  |  |  |  |  |  |  | 0.041 |  |  |
| R Squared |  |  | 0.1867 |  |  |  |  |  |  |  |  |  |  |  | 0.1579 |

* ρ <0.001
